# Feasibility of sonographic access to the cricothyroid membrane in the presence of a rigid neck collar in healthy Chinese adults: A prospective cohort study

**DOI:** 10.1002/ajum.12187

**Published:** 2019-10-17

**Authors:** Lok Yu Wong, Marc L. C. Yang, Hei Jim Leung, Chi Shing Pak

**Affiliations:** ^1^ Accident and Emergency Department Queen Elizabeth Hospital 30 Gascoigne Road Kowloon Hong Kong

**Keywords:** airway, emergency department, pocus, trauma, cricothyroidotomy

## Abstract

**Objectives:**

(1) To study the dimensions of cricothyroid membranes (CTMs) in healthy Chinese adults in two neck positions, one with rigid neck collar (RNC) and neck extended by ultrasonography (USG). (2) To evaluate how body habitus and neck positions may affect the access time of CTMs, and thus the feasibility for ultrasound‐guided cricothyroidotomy.

**Methods:**

We scanned 39 adult staff of a local emergency department. Their CTMs were measured by two emergency physicians (EP) separately. The subjects' gender, weight, height, age, neck circumferences and BMI were collected. Image qualities (graded in ‘inadequate, adequate and good’) and image acquisition time of the CTMs were also recorded to ascertain proper CTM measurements.

**Results:**

The mean depth of the CTM (neck extended) was 5.6 mm, and the standard deviation (SD) was 1.52. The mean depth (with RNC) was 5.97mm with SD 1.61. The mean length of the CTM (mm ± SD) with the neck extended and with the RNC was 10.5 ± 2.15 and 9.97 ± 2.24, respectively. The median image acquisition time for neck extended was 6.36s with interquartile range (IQR) of 2.32–8.4 s, while for RNC the median time was 5.60 s (IQR = 3.71–7.49; P = 0.539). Image acquisition time between the first and the second sonographers was similar. All subjects’ CTM could be identified readily by USG.

**Conclusions:**

The CTM can be located quickly and reliably by bedside USG, even in overweight/obese persons with or without an RNC in place. We recommend that further study on the feasibility of bedside cricothyroidotomy with RNC kept on should be explored.

## Introduction

### Background

Ultrasound scan is non‐invasive, produces no ionising radiation and is relatively easy to learn. As a result, ultrasonography has gained popularity in recent years as a diagnostic tool and an aid in image‐guided interventions in accident and emergency departments (AED). Ultrasound scans of anterior neck structures are still uncommonly performed in AEDs in Hong Kong. Although it is operator‐dependent, desirable image capture can often be achieved on superficial structures such as the upper airway through the anterior neck approach. A local study at a major local hospital showed it was quick and straightforward to locate the epiglottis of healthy Chinese adults with little interobserver difference.^1^

Securing airways is often required in EPs’ daily practice.^2^, ^3^ In cases of difficult airways, whether predicted or unpredicted, EPs may need to ventilate the patients through surgically created access on the cricothyroid membranes. Cricothyroidotomy is an uncommon yet life‐saving procedure performed on patients with difficult airways. Major guidelines for emergency medicine and anaesthesiology recommend that, as a last resort, in both anticipated and unanticipated difficult airway scenarios, a surgical airway through the anterior neck is indicated to save the patients’ lives in ‘cannot intubate, cannot ventilate’ situations.^4^

The CTM is traditionally located through palpation of the thyroid cartilage and cricoid cartilage. ‘Laryngeal handshake’ is an example of such a technique. An incision is made on the groove between the two cartilaginous prominences to reach the CTM.^5^ However, in unexpected difficult airway situations, the CTM can be challenging to locate.^6^ Patient factors such as previous neck surgery, radiotherapy, obesity and neutral neck position (as in trauma patients with a rigid neck collar (RNC) being put on) further exacerbate the hurdles. In a recently published review article, the authors compared studies on success rates on the location of the CTM by palpation among people with different body habitus and neck positions, and they found it was not always possible to succeed even in non‐obese persons, and the success rates on obese patients were even lower (72% non‐obese males vs. 39% obese males).^7^ Emergency airway is in general difficult to establish. One study quoted 36% success rate on emergency airway access performed by anaesthesiologists,^8^ and another study reported an even lower success rate on obese persons (0–39%).^7^

With regard to this problem, research in emergency medicine and anaesthesiology has revealed promising potentials in the sonographic location of the CTM and ultrasound‐guided cricothyroidotomy. Current evidence has shown that the CTM can be located quickly and accurately by sonographers. An early study showed that bedside ultrasound scans performed by emergency physicians were effective in locating the CTM relatively quickly (mean = 24.32 ± 20.18 s).^9^ A cadaveric research studied cricothyroidotomy under sonographic guidance and concluded that the procedure could be done on average under 30 s with a very low failure rate (1 out of 21 or 4.7 %).^10^ Another study which employed a model simulating a patient with difficult anterior neck anatomy was able to prove that anaesthesiologists performed better in establishing a cannula tracheotomy with ultrasound guidance versus the conventional unguided method.^11^ Ultrasound scans can also give precise dimensions of the CTM, even in paediatric patients. The height of CTM in paediatric patients was shown to be accurately measured by anaesthesiology trainees using ultrasound scans against magnetic resonance imaging. The correlation coefficient was 0.98 (95% CI 0.95–0.99, P < 0.0001).^12^

For adult major trauma patients, 2–5% of them may have associated cervical spine injury, of which more than 10% of such injuries may be unstable.^13^ In order to minimise secondary cervical spinal injuries, for patients with a suspected cervical spine injury, RNCs are typically applied before or on arrival to the emergency departments. Oral or nasoendotracheal intubation may be contraindicated for those with maxillofacial injuries, and an emergency surgical airway such as cricothyroidotomy may be indicated.^14^ Current methods of performing an emergent surgical airway may involve the identification of the CTM with the neck in neutral position with manual in‐line stabilisation (MILS) of the neck.^13^ However, immobilisation by MILS is not perfect, which is operator‐dependent after all. It may exacerbate cervical spine injury as MILS may be associated with distraction and subluxation of the cervical spine.^15^ This study explores the practicability of locating the CTM, a crucial step for the establishment of a cricothyroidotomy, by ultrasound scan with an RNC being applied on the subjects.

There are currently few similar studies done on Chinese in AEDs on sonographic measurements of the CTM and probably none with an RNC being kept on during the scan. This study aims to measure the length, width and depth of the cricothyroid membrane by ultrasonography with subjects’ necks being placed in extended positions or neutral position with an RNC, and to study how those measurements relate to different body habitus in healthy Chinese adults. This paper is probably the first study to report the sonographic dimensions of CTM in healthy Chinese adults and the first to do so with an RNC in place during the scan.

### Objectives

(1) To study the range of dimensions of CTMs in healthy Chinese adults in two different neck positions (with RNC and neck extended) by ultrasonography. (2) To evaluate how body habitus may affect the access time of CTMs in either neck position and thus the feasibility for ultrasound‐guided cricothyroidotomy.

## Methods

### Study design and setting

The study was a prospective matched cohort study conducted in the Accident and Emergency Department of Queen Elizabeth Hospital in Hong Kong. The Queen Elizabeth Hospital is a regional tertiary and quaternary referral centre, and trauma centre. It has an average daily emergency attendance of 500 patients. Recruitment and data collection were conducted between 1 March and 31 May 2019. This study was reported in compliance with the Strengthening the Reporting of Observational Studies in Epidemiology (STROBE) statement.^16^

Ethical approval was granted by the Research Ethics Committee of the Kowloon East and Central clusters of the Hospital Authority (Ref.: KC/KE‐18‐0301/ER‐1).

### Participants

Convenience sampling of Chinese adults who were staff in the Accident and Emergency Department was performed. Exclusion criteria included (i) extensive cervical spine diseases, (ii) structural upper airway diseases (e.g. laryngeal cancer), (iii) previous operations to the larynx and (iv) anterior neck masses due to various causes. Each subject was matched onto himself/herself for comparison of the effect of neck positions. Each participant was given information on the study, and written consent has been obtained.

### Sample size

Assuming the clinically significant difference in the depth, length and width caused by the different positions of the neck is more than 1mm, a sample size of 32 in each group will be needed.^17^ Forty‐four persons without any exclusion criterion had agreed to enrol in the study.

### Variables and measurements

**Figure 1:**
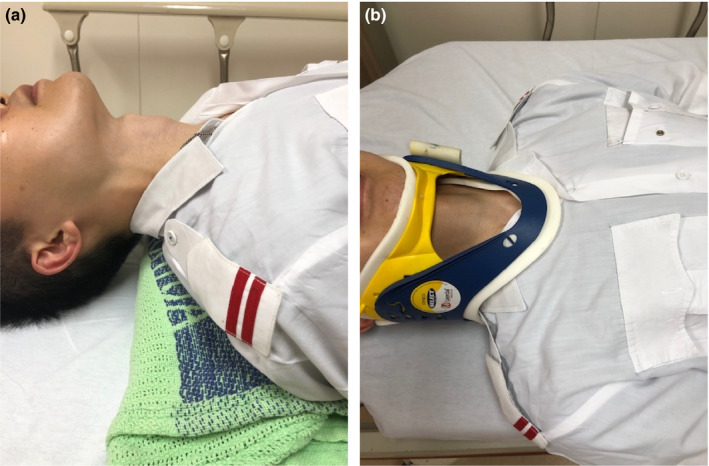
(a) Neck in extension. (b) Neck with an RNC applied.

Ultrasound scans were done by utilising the L10‐5v linear probe of the Siemens Acuson P500 machine with the probe setting: ‘Small Parts’ chosen. This machine setting was kept the same for all scans. The subjects were scanned first with their necks extended (Figure 1a) and then in the neutral position with an RNC (Adult Laerdal™ Stifneck® Select™ Collar) applied (Figure 1b). The scans all started with the transverse approach with the probe being placed just caudal to the prominence of the thyroid cartilage (or mid‐point of the anterior neck if the prominence was not well seen). After a still image was captured, the probe was then turned 90 degrees for the longitudinal view. The sonographers were allowed to examine the patients’ necks prior to commencing the scans. The scanning was done in actual clinical areas of the AED alongside other clinical activities; hence, the findings of this study (especially image quality and scanning time) would be as close to real‐world figures as possible.

All the subjects were scanned by two sonographers: one EM trainee and the other an EM specialist. Time to the acquisition of image is defined as the time between skin contact by the probe and a standard still image of the CTM being acquired with a transverse view. The images would then be graded as ‘inadequate’, ‘adequate’ and ‘good’, to ensure proper measurement of CTM parameters.

**Figure 2:**
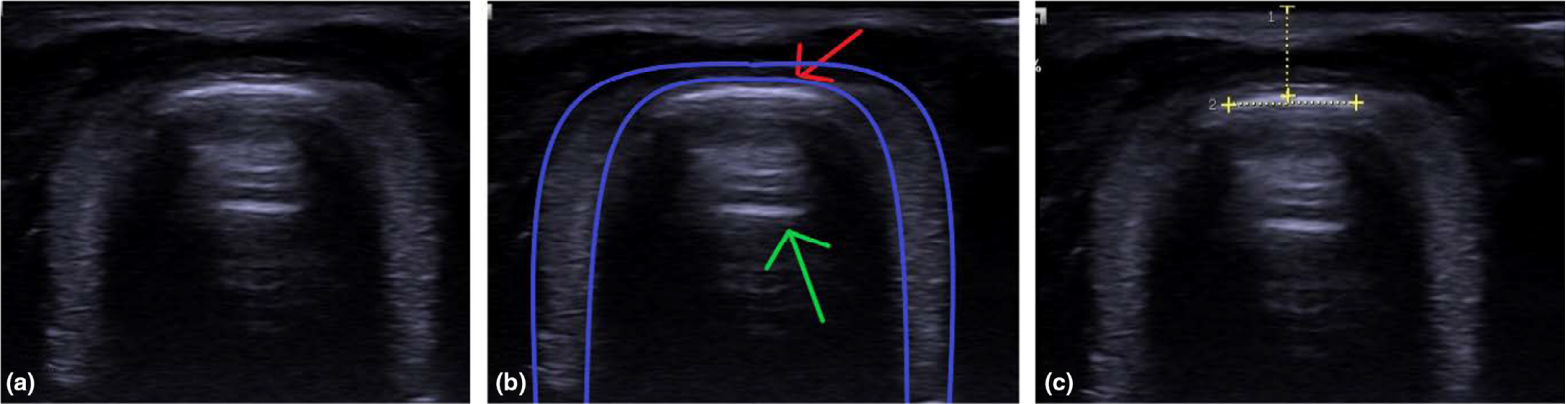
(a) Ultrasound image showing the transverse view of the CTM. (b) The CTM is seen as a white horizontal line formed at the mucosa–air interface (red arrow). White lines deep to the CTM are reverberation artefacts (green arrow). (c) Depth and width of the CTM are measured as shown.

**Figure 3:**
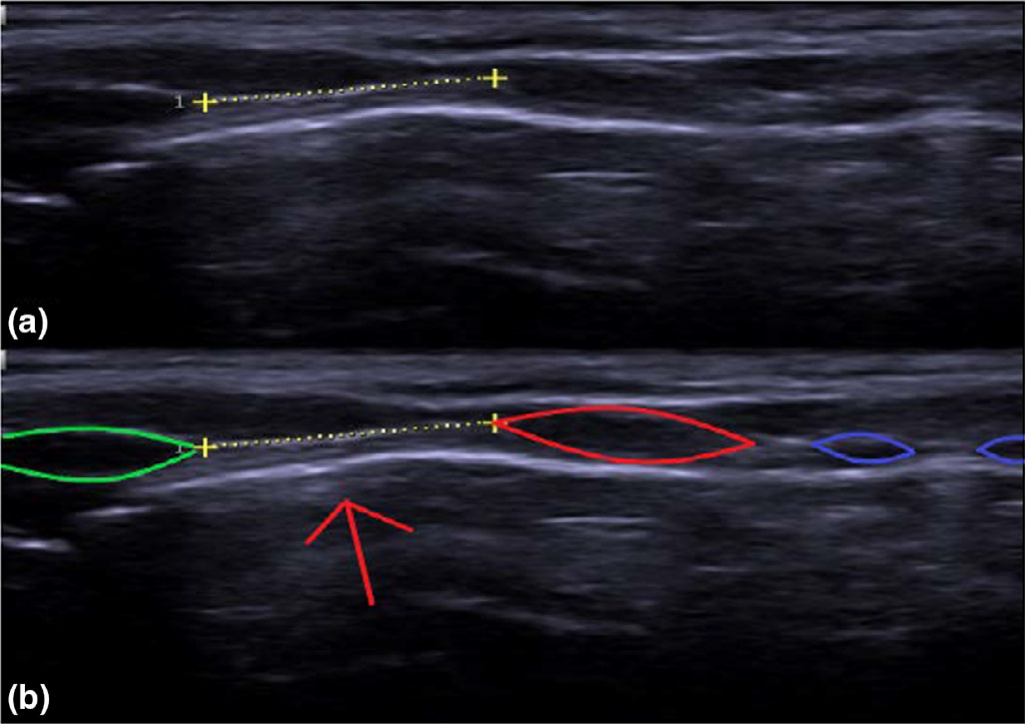
(a) Ultrasound image showing the longitudinal view of the CTM. (b) The CTM is seen as a white horizontal line formed at the mucosa–air interface (red arrow). Tracheal rings are shown in blue ovals. Cephalically, the CTM is bound by the inferior edge of the thyroid cartilage (green oval), while caudally, the CTM is bound by the superior border of the cricoid cartilage (red oval). The length of the CTM is measured between these borders.

An ‘inadequate’ image reveals the location of the CTM, but the neighbouring structures are not clear enough for precise measurements of all the parameters described above. A ‘good’ image reveals the CTM and outlines of neighbouring structures clearly. An ‘adequate’ image reveals all relevant anatomy with less clarity than that of a ‘good’ image. A ‘good’ standard transverse and longitudinal view is shown (Figures 2 and 3). Two authors assessed image qualities independently with disagreement adjudicated by a third author. Measurement of the CTM dimensions and image acquisition times from both sonographers were averaged.

The measurements and image acquisitions were made independently to reduce biases. Image qualities were assessed after completion of all data collection, without information on the individual biological characteristics to reduce bias.

The biological characteristics of the subjects: gender, age, height, weight, neck circumferences, were also measured at the time of image acquisition. Neck circumference was measured by a soft tape measure at the level of the CTM (if CTM was palpable or just below the thyroid cartilage if the CTM was not easily palpable). Measurement of biological parameters was conducted by an author and assistant who were not involved in ultrasound scanning to reduce bias.

### Statistical methods

Continuous variables (e.g. age, height, CTM measurements) were assessed by the Shapiro–Wilk test for normality. These were then presented as mean or median with standard deviation (SD) or interquartile range (IQR). The measurements of the CTM in either neck position were reported as an average of the data obtained by the two sonographers.

The effect of neck position and presence of neck collar on the dimensions of the CTM were compared with paired‐sample t‐test and Wilcoxon sign‐rank test where appropriate. The relations between the depth and length of the CTM and the aforementioned biological factors were analysed by multiple linear regression models in a stepwise manner. All P values were two‐tailed, and 0.05 was picked as the significance level.

All statistical analyses and data processing were performed by Microsoft® Office Excel 2016 and IBM SPSS® Statistics version 25.

### Ethical approval

Ethical approval was granted by the Research Ethics Committee of the Kowloon East and Central clusters of the Hospital Authority (Ref.: KC/KE‐18‐0301/ER‐1). This study was completed in accordance with the Helsinki Declaration.

## Results

### Participants and descriptive data

Forty‐four Chinese adults were recruited for the study. After data collection and preliminary analysis, five subjects were excluded because of technical errors which resulted in incomplete image sets. The data of thirty‐nine subjects underwent statistical analysis.

The biological characteristics of the subjects are shown in Table 1. The median age (IQR) of the subjects was 32 years old (22–42), which was typical of a working‐age cohort. 77% of them were male. Their median weight (IQR) was 65kg (55.5–74.5), while the mean height (SD) was 168.8 cm (± 8.2). The mean neck circumference (SD) was 35cm (±4.3). The mean BMI (SD) was 23.2 (±4). The overall characteristics of the subjects suggest that the study subjects encompass a broad spectrum of body habitus.

**Table 1:** The biological characteristics of the subjects.

Variables	Whole cohort (N = 39)
Age (median/IQR)	32 (22–42)
Male (%)	30 (77%)
Weight/kg (median/IQR)	65 (55.5–74.5)
Height/cm (mean/SD)	168.8 ± 8.2
BMI/kg/m^2^ (mean/SD)	23.2 ± 4
Neck circumference/cm (mean/SD)	35 ± 4.3

CTM, cricothyroid membrane; BMI, body mass index; SD, standard deviation; IQR, interquartile range.

### Main results

The dimensions of the CTM (mean ± SD) with the neck extended and with the RNCs as well as time to image acquisition (median/IQR) are shown in Table 2. The CTM depth (mm) with the neck extended and the RNC was 5.6 ± 1.52 and 5.97 ± 1.61, respectively, while the CTM length (mm) was 10.5 ± 2.15 and 9.97 ± 2.24, respectively. The differences in depth and length were statistically significant. On the other hand, the CTM width (mm) with the neck extended and the RNC was 7.35 ± 1.62 and 7.20 ± 1.49, respectively; however, the differences did not reach statistical significance.

**Table 2:** The CTM dimensions and image acquisition times.

	With neck extended	With neck collar	P‐value
CTM depth/mm (mean ± SD)	5.6 ± 1.52	5.97 ± 1.61	0.04*
CTM length/mm (mean ± SD)	10.5 ± 2.15	9.97 ± 2.24	0.028*
CTM width/mm (mean ± SD)	7.35 ± 1.62	7.20 ± 1.49	0.544*
Time to image acquisition/seconds (median/IQR)	6.36 (2.32–8.4)	5.60 (3.71–7.49)	0.539#

CTM, cricothyroid membrane; SD, standard deviation; IQR, interquartile range.

* Paired‐sample t‐test.

^#^ Wilcoxon sign‐rank test.

The depth of the CTM was also found to correlate positively with body weight and negatively with body height, as shown by the linear regression model in Table 3. For those with RNC, for every 1 kg increase in body weight, the depth of the CTM would increase by 0.074mm. In addition, for every 1 cm increase in body height the depth would decrease by 0.08mm. For those with neck extension, for every 1 kg increase in body weight, the depth of the CTM would increase by 0.08mm. Likewise, for every 1 cm increase in body height, the depth would decrease by 0.09 mm.

**Table 3:** Linear regression results.

Linear regression						
	Depth of CTM		Length of CTM		Width of CTM	
	Coefficients	p‐value	Coefficients	P‐value	Coefficients	P‐value
Neck extended						
Age	Not in model		Not in model		Not in model	
Male	Not in model		3.6	<0.0005	Not in model	
Weight/kg	0.08	<0.0005	Not in model		Not in model	
Height/cm	−0.09	<0.0005	Not in model		Not in model	
Neck Circumference/cm	0.12	0.09	−0.2	0.03	Not in model	
R square of model	0.74		0.298			
With neck collar						
Age	Not in model		Not in model		−0.5	0.014
Male	Not in model		2.92	<0.0005	1.22	0.02
Weight/kg	0.074	<0.0005	Not in model		Not in model	
Height/cm	−0.08	0.001	Not in model		Not in model	
Neck circumference/cm	0.146	0.005	Not in model		Not in model	
R square of model	0.691		0.31		0.268	

It was also noteworthy that the length of CTM was found to be positively correlated with male gender in either neck position (P < 0.0005). Although R square was low (neck extended = 0.298; RNC = 0.31), this finding concurred with that of the study which employed computer tomography scans to measure CTM lengths.^18^

### Other analyses

The average time to image acquisition (IQR) with the transverse view was similar (neck extended = 6.36 s (2.32–8.4) vs. RNC = 5.60 s (3.71–7.49); P = 0.539), and most of the images could be acquired within 10 s. It was found that the CTM was shallower (P = 0.04) and longer (P = 0.028) on average when the neck was extended, agreeing with the current understanding that an extended neck would provide the best access to the CTM.^7^ Furthermore, the differences in time to image acquisition between the first and second sonographers were also analysed. Friedman’s two‐way ANOVA was employed, and it showed that there was no significant time difference between the two sonographers (P = 0.514).

Ultrasonography can also be utilised to identify the CTM reliably, even when an RNC is worn. Of all the 156 scans (i.e. n = 39 × 4) done by both sonographers, only ten image series were deemed ‘inadequate’ (6.4%). Nine of these 10 ‘inadequate’ series were reported in those wearing an RNC. Image quality was worse in the RNC group because the collar itself posed a physical barrier and hence decreased the manoeuvrability of the ultrasound probe. The challenge was more pronounced in locating the inferior border of the thyroid cartilage.

## Discussion

### Key results

The neck position and the presence of an RNC affect the accessibility and the image quality of the CTM; nonetheless, image acquisition is straightforward.

As shown in Table 2, neck positions did affect the dimensions of the CTM. Neck extension was associated with a shallower (5.6 mm ± 1.52 vs. 5.97 mm ± 1.61; P = 0.04) and longer (10.5 mm ± 2.15 vs. 9.97 mm ± 2.24; P = 0.028) CTM compared with those with an RNC applied. Therefore, percutaneous access through the CTM would be easier with neck extended. However, in cases where spinal immobilisation must be maintained by an RNC, percutaneous access is still feasible as the depth and length of the CTM in this neck position are not impractically deeper and shorter.

This study also revealed that the female gender and being shorter were both associated with a shorter CTM while being heavier was associated with a deeper CTM (Table 3). Knowledge of the relations between biological factors and CTM dimensions would allow EPs to identify patients with potential difficult surgical airway access early.

This study was able to show bedside ultrasound scans could be used to quickly locate the CTM, even with an RNC in place. The median times for image acquisition with the neck extended and with the RNC were 6.36 s (2.32–8.4) and 5.60 s (3.71–7.49), respectively, and the difference was not statistically significant. Although an RNC may pose an obstacle for an ultrasound scan as it restricts the working space for the probe over the anterior neck, the CTM can still be rapidly seen in a matter of seconds.

Ultrasonography is a reliable method to identify the CTM, even for operators with different levels of experience in ultrasonography. CTMs of all the study subjects could be located clearly in the standard transverse view by every sonographer in this study. All ‘inadequate’ images that were included in the analysis were deemed so due to the difficulty to determine the inferior border of the thyroid cartilage in the longitudinal view, making measurements of the CTMs’ length challenging. The vast majority of the images in both groups were deemed ‘adequate’ or ‘good’.

Furthermore, the ten inadequate image series (6.4% of all scans) belonged to 6 participants, of which only one was obese (BMI = 28.7 kg/m^2^).^19^ The BMI of the rest in this group was 18‐22.6. On the other hand, there were overall six overweight subjects (BMI 23–24.9) and 11 obese subjects (BMI ≥ 25) included for analysis, suggesting that ultrasound scans performed well in locating overweight and obese subjects' CTMs. Therefore, it can be extrapolated that extensive training and experience in diagnostic ultrasonography are not needed to locate the CTM in either neck position, with or without an RNC and even in overweight and obese subjects.

Cricothyroidotomy rates in emergency departments are suggested to be around 2–3% but is reported to be up to more than 12% in some research.^13^ Since EPs may encounter unexpected difficult airway situations from time to time, it is reasonable to suggest that, before initiation of airway management, the CTM should routinely be assessed and located,^20^ especially in those whose CTMs are known to be difficult to locate by palpation alone (e.g. obese patients^7^ and those with poorly defined surface neck anatomy).^20^ One recent randomised control trial reported a 10‐fold increase in accuracy of CTM location by ultrasound vs palpation.^21^ Better preparations would not just lead to an ease of mind but could potentially lead to a better outcome. Given the encouraging evidence in the versatility of bedside ultrasonography in CTM assessment and the availability of ultrasound machines in AEDs, all emergency department doctors should be trained and encouraged to perform such scans. Further studies may be needed to investigate the practicality of performing an ultrasound‐guided cricothyrotomy with an RNC put on, which may potentially save time and minimise cervical spine injury by maintaining immobilisation. Moreover, the trauma team member who is assigned initially to perform MILS can be directed to assist in other life‐saving tasks.

#### Limitations

This study has certain limitations, as convenience sampling may give rise to selection bias and sampling error. Firstly, the data might be biased as there was more male than female (23%). Moreover, the subjects were relatively young (aged 21–65 years old). The results may not be suitable to be generalised to older populations. Nonetheless, the study design is still reasonable for generating primary data for a topic that has not been studied extensively before.

Moreover, the time to image acquisition was found to be shorter in those with an RNC, though the difference was not statistically significant (P = 0.539). It could be attributed to the scan sequence, in which the subjects were always scanned with neck extended first, then with an RNC. As a result, the sonographer would always be scanning the subject the second time when the neck collar was put on, eliminating the novelty effect.

Finally, ultrasonography is operator‐dependent. Different operators may exert a different degree of pressure on the skin and soft tissue underneath the probe, which may affect the depth of the CTM measured. This effect was mitigated by scanning the subject twice by different operators. 

The current study was not conceived and powered to investigate the effect on image quality and image acquisition time incurred by the presence of an RNC.

## Interpretation and Conclusions

The CTM can be located quickly and reliably by bedside ultrasound scans, even in overweight and obese persons with or without an RNC in place. The extended neck position may improve any percutaneous access to the CTM by lengthening and reducing the depth of the CTM, in keeping with current knowledge. However, we are also able to demonstrate the relative ease of identifying the CTM by ultrasonography with the RNC being in place throughout the procedure. We recommend that further study on the feasibility of performing bedside cricothyroidotomy with the RNC kept on should be explored.

## Generalisability

The findings of this study should be readily generalisable to any urban AED with access to bedside ultrasound on patients with normal neck anatomies.

## Authorship statement

LYW, MY and CSP conceived the study. LYW and MY were responsible for literature review, ethical approval, statistical analysis and the first draft of the manuscript. LYW, MY, HJL and CSP were responsible for the synthesis of the original data set and revision of the manuscript.

## Funding

The authors received no financial support for the research, authorship and/or publication of this article.

## Conflict of interest

The authors have no conflict of interest to declare.

## Informed consent

Informed consent was sought, and each participant has received an information sheet about the study.

## Human rights

Human rights is respected. Ultrasound scans that the subjects have received are well known to be safe. No information identifiable to individuals has been collected and stored.

## Data Availability

The data sets generated and/or analysed in the current study are available. They have been submitted alongside other documents.
